# A non-linear relationship between triglyceride glucose waist circumference and nonalcoholic fatty liver disease in a Japanese population: a secondary analysis

**DOI:** 10.3389/fendo.2023.1188214

**Published:** 2023-07-07

**Authors:** Xiaojie He, Xinyue Huang, Yafang Qian, Ting Sun

**Affiliations:** ^1^ Department of Health Management Center, The Second Affiliated Hospital, Zhejiang University School of Medicine, Hangzhou, Zhejiang, China; ^2^ School & Hospital of Stomatology, Wuhan University, Wuhan, China

**Keywords:** nonalcoholic fatty liver disease, insulin resistance, triglyceride, fasting blood glucose, triglyceride glucose waist circumference

## Abstract

**Introduction:**

Nonalcoholic fatty liver disease (NAFLD) is a common metabolic disorder associated with insulin resistance (IR). Triglyceride glucose waist circumference (TyG-WC) is a novel index of IR that reflects both visceral fat and hepatic steatosis. However, it is not known whether TyG-WC and NAFLD exhibit a nonlinear relationship in Japanese subjects with normal plasma glucose level. Thus, we examined the relationship between TyG-WC and NAFLD, in addition to determining the threshold level of TyG-WC associated with NAFLD.

**Methods:**

A secondary analysis was performed based on a previous study that extracted medical examination records from Murakami Memorial Hospital between 2004 and 2015 in order to detect chronic diseases and their risk factors. TyG-WC was determined at baseline. NAFLD is the dependent variable. Univariate and multivariate logistic regression models were used to evaluate the risk of NAFLD incidence. Based on the smoothing plot, a two-piecewise linear regression model was used to examine the threshold effect of TyG-WC on NAFLD. A subgroup analysis was carried out in order to study other factors that may influence the association between TyG-WC and NAFLD.

**Results:**

14,280 met the criteria for inclusion in the current secondary analysis. The adjusted OR (95% CI) for NAFLD in all subjects was 1.007 (95% CI 1.006–1.009, P < 0.001). The relationship between TyG-WC and NAFLD in Japanese subjects with normal plasma glucose level is nonlinear. TyG-WC is positively associated with NAFLD when TyG-WC is ranged between 480 and 800. In subgroup analyses, there was a significant interaction between BMI and TyG-WC associated NAFLD risk (P for interaction <0.001).

**Discussion:**

The relationship between TyG-WC and NAFLD is nonlinear. TyG-WC is positively associated with NAFLD when TyG-WC is ranged between 480 and 800. There is potential clinical significance for the TyG-WC in identifying groups at high risk for NAFLD in subjects with normal plasma glucose level.

## 1 Introduction

NAFLD is characterized by a variety of histopathologic findings, ranging from steatosis to steatohepatitis, fibrosis, and cirrhosis (1). A number of factors contribute to NAFLD, including metabolic syndrome, obesity, insulin resistance, and hyperlipidaemia (2, 3). Along with the global obesity-related metabolic syndrome epidemic, NAFLD prevalence is increasing (4). Approximately 25% of the population worldwide suffers from NAFLD, ranging from 13% in Africa to 42% in southeast Asia (5, 6). Metabolic dysfunction-associated liver disease (MAFLD) is characterized by metabolic dysregulation and overlaps with other liver diseases, according to an expert panel recently (7). There has, however, been a lack of widespread adoption of the new definition. In order to predict and diagnose NAFLD early, accurate non-invasive methods must be investigated, as it is normally asymptomatic until the advanced stages (8, 9).

Regardless of whether metabolic syndrome is present or absent, there is evidence that IR contributes significantly to the development of NAFLD (10, 11). The triglyceride and glucose index (TyG) has been proposed as an effective alternative to IR. This combines fasting plasma glucose (FPG) with fasting triglyceride (TG) (12). TyG levels have been associated with NAFLD incidence in many studies (13). The combination of TyG-related parameters and obesity indices may be more reliable for identifying patients than TyG alone, according to emerging research (14–17). Evidence indicates TyG-waist circumference (TyG-WC) is a superior predictor of insulin resistance than TyG alone (14, 18, 19). TyG-WC has been shown to be related to NAFLD among Iranians in a cross-sectional study (20).

In Japanese people, however, it is not known whether TyG-WC is associated with NAFLD. Further, it is not clear whether TyG-WC and NAFLD exhibit a nonlinear relationship. In this study, we examined the relationship between TyG-WC and NAFLD in Japanese subjects with normal plasma glucose level, in addition to determining the threshold level of TyG-WC associated with NAFLD.

## 2 Materials and methods

### 2.1 Data source

A public database called Datadryad.org, where investigators can reanalyse data from previous studies, was used for this study. The research cites Okamura et al.’s data packets (21). A second analysis was performed based on previous research that aims to detect chronic diseases and their risk factors. Previous study extracted cases from Murakami Memorial Hospital’s medical examination program between 2004 and 2015, then, a follow-up study was carried out on incident type 2 diabetes and fatty liver.

This study adopted a cross-sectional design and its exclusion criteria are as follows: 1) viral hepatitis (determined by hepatitis B antigen and hepatitis C antibody measurements)(N=416); 2) drinking excessively: males 210 grams per week or females 140 grams per week (N=1923) (22); 3) data with incomplete covariables (N = 873); 4) T2DM or fasting plasma glucose over 6.1 mmol/L at baseline-examination (N = 1131); 5) any medication usage (N = 2,321). Informed consent was not required because the data had been de-identified. Murakami Memorial Hospital’s ethics committee approved the previous study (21). As a result, there was no need for an additional ethical approval for this study. It followed the Declaration of Helsinki.

### 2.2 Collection of data

As mentioned previously, a self-administered questionnaire was adopted to collect clinical baseline information (21). A comprehensive list of demographic information, anthropometric and clinical measurements or lifestyle characteristics, including sex, age, height, weight, WC, systolic blood pressure (SBP), diastolic blood pressure (DBP), drinking habit, and smoking habit. Exercise habit is defined as exercising more than once a week; Smoking status was divided into nonsmokers, former smokers, and current smokers based on smoking history. Drinking status was classified into three groups based on alcohol consumption: minimal or no consumption, 40 grams or less per week; light, 40-140 grams per week; moderate, 140-210 grams per week. Haematological indicators were tested after fasting, including haemoglobin A1c (HBA1c), total cholesterol (TC), triglyceride (TG), FPG, gamma-glutamyl transferase (GGT), aspartate aminotransferase (AST), alanine aminotransferase (ALT), and high-density lipoprotein cholesterol (HDL-C). According to previous studies (14, 23), TyG = Ln [TG (mg/dL) × FPG (mg/dL)/2], TyG-WC = TyG × WC (cm).

### 2.3 NAFLD diagnosis by abdominal ultrasound

A trained technician performed abdominal ultrasonography to diagnose fatty liver. Without consulting any other information about the participants, the gastroenterologist diagnosed fatty liver based on the images. Among the four known criteria (vascular blurring, hepatorenal echo contrast, deep attenuation and liver brightness), participants with hepatorenal echo contrast and liver brightness were diagnosed with fatty liver (24).

### 2.4 Statistical analysis

For all statistical analyses, EmpowerStats (www.empowerstats.com , X&Y Solutions, Inc., Boston, MA) and R (http://www.R-project.org, The R Foundation) platforms were used. Categorical variables were expressed as the frequency (percentage).

Normal distribution continuous variables were expressed as mean ± standard deviation (SD), and unnormal distribution continuous variables as median (quartile 1-quartile 3). By using Kruskal Wallis H (variables with non-normal distribution) or one-way ANOVA (variables with normal distribution) or chi-squared(categorical) tests, differences among TyG-WC groups were evaluated. A two-tailed *P* value of 0.05 was used to determine statistical significance. NAFLD risk factors were determined using multivariate and univariate logistic regression models. We also explored the relationship between TyG-WC and NAFLD according to gender. Initially, all variables were analysed using univariate analysis.

Afterwards, variables with clinical significance and variables with statistical significance in univariate analysis (*P* < 0.05) were included in the multivariate analyses. In order to evaluate the collinearity of all explanatory variables, a correlation matrix was used. Variance inflation factor (VIF) based multiple regression model was used to assess collinearity (25). The **Additional File Table S1** illustrates the presence of collinearity when the VIF is greater than 5. Four different models were built: Model 1, without covariate adjustment; Model 2, adjusted for age, smoking status, habit of exercise, SBP; Model 3, adjusted for Model 2+HbA1C, FPG, TG, TC, HDL, GGT, ALT, AST, and Model 4, adjusted for Model 3+BMI. We selected these confounders on the basis of their associations with the NAFLD or a change in effect estimate of more than 10% (26).

The data analysis results were verified by converting TyG-WC according to triquantiles and examining the possibility of nonlinearity, and calculation of the *P* value for the trend was carried out. The threshold effect of TyG-WC on NAFLD was examined using a two-piecewise linear regression model based on the smoothing plot. In order to determine the threshold level of TyG-WC at which the association between TyG-WC and NAFLD changed and became noticeable, a recurrence method was employed. Detecting the maximum model likelihood was based on moving the inflection point along a predefined interval. Logistic regression model was used for subgroup analysis. To test the subgroup effect modification, interaction terms were used between subgroup indicators, followed by likelihood ratio tests.

## 3 Results

### 3.1 Subjects description

**Figure 1:**
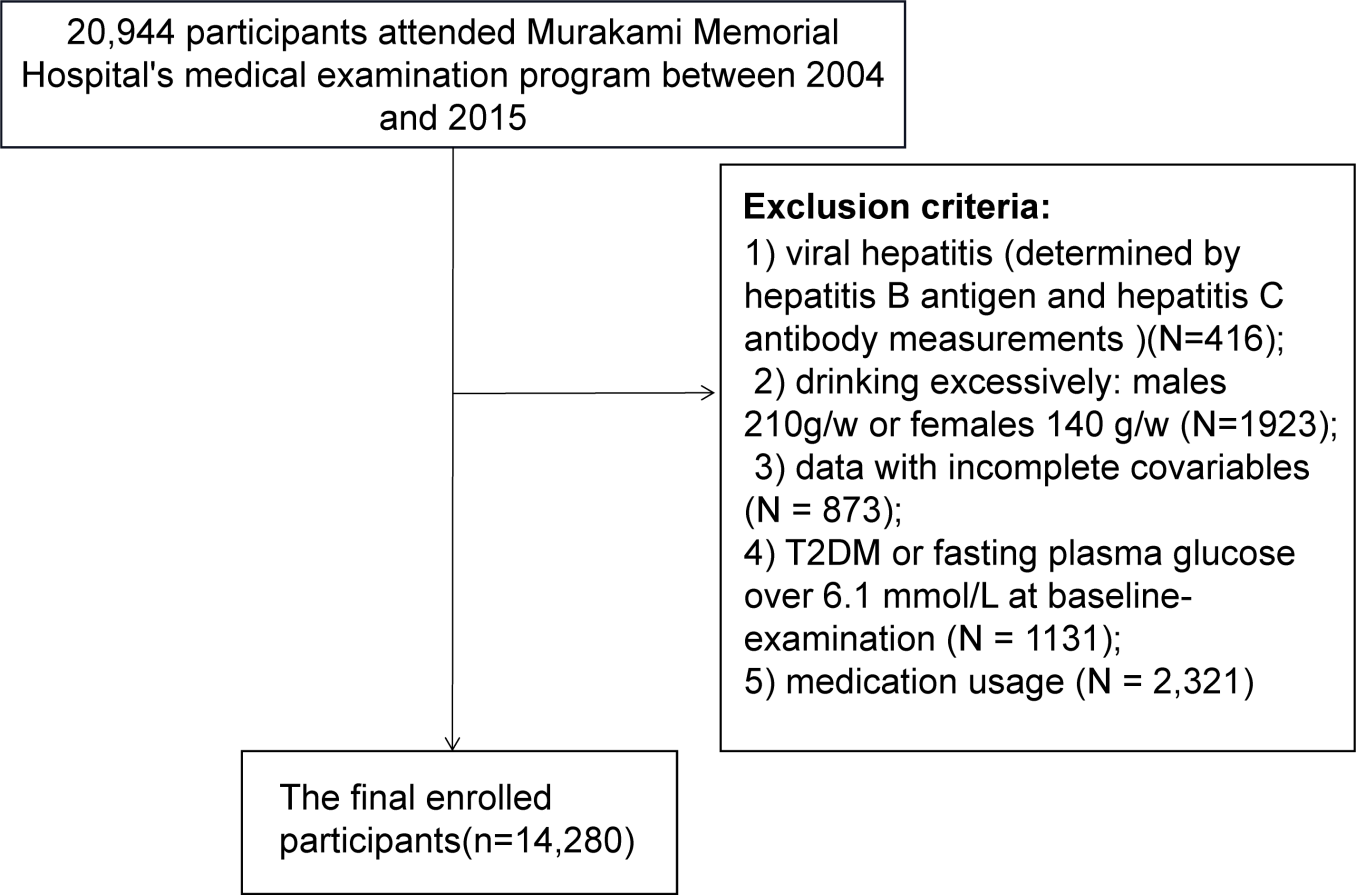
Flow chart.

In the previous study, 20,944 subjects were recruited. Only 14,280 met the criteria for inclusion in the current secondary analysis (**Figure 1**). The average age of the subjects was 44 ± 9 years, and 54.51% were men. Subject baseline characteristics are listed in **Table 1**. TyG-WC group (T3) individuals were usually older and had higher BMI, WC, SBP, DBP, ALT, AST, GGT, TG, TC, HbA1C, TyG values than TyG-WC group (T1). Comparatively, HDL-C values for T3 groups were lower than those for T1 groups. Additionally, the prevalence of NAFLD gradually increases as the value of TyG-WC increases (T1: 0.626% vs. T2: 9.226% vs. T3: 44.864%).

**Table 1:** Characteristics of the participants by triquartile grouping of TyG-WC.

TyG-WC	T1 (339.450-559.133)	T2 (559.145-660.110)	T3 (660.112-1097.184)	*P*-value
N	4955	4769	4556	
Age, years	41.055 ± 8.467	44.260 ± 8.877	45.466 ± 8.739	<0.001
BMI, kg/m2	19.550 ± 1.786	21.845 ± 1.897	25.040 ± 2.797	<0.001
Weight, kg	50.467 ± 6.185	59.828 ± 7.252	71.436 ± 9.854	<0.001
WC, cm	67.329 ± 4.645	76.223 ± 4.138	85.811 ± 6.340	<0.001
SBP, mmHg	106.176 ± 12.313	114.288 ± 13.169	122.084 ± 14.534	<0.001
DBP, mmHg	65.717 ± 8.559	71.145 ± 9.357	77.037 ± 10.020	<0.001
ALT, IU/L	13.000 (11.000-17.000)	16.000 (13.000-21.000)	23.000 (17.000-32.000)	<0.001
AST, IU/L	16.000 (13.000-19.000)	17.000 (14.000-20.000)	19.000 (16.000-23.250)	<0.001
GGT, IU/L	12.000 (10.000-14.000)	15.000 (11.000-20.000)	21.000 (15.000-31.000)	<0.001
TG, mg/dL	40.000 (30.500-54.000)	64.000 (50.000-82.000)	112.000 (84.000-154.000)	<0.001
TC, mg/dL	187.155 ± 31.071	197.971 ± 32.589	210.234 ± 33.052	<0.001
HDL, mg/dL	65.758 ± 14.560	56.789 ± 13.751	45.959 ± 10.837	<0.001
HbA1C, %	5.118 ± 0.302	5.175 ± 0.310	5.246 ± 0.338	<0.001
TyG-WC	502.080 ± 38.700	608.815 ± 28.657	739.364 ± 64.803	<0.001
TyG	7.466 ± 0.448	7.999 ± 0.382	8.620 ± 0.472	<0.001
Gender				<0.001
Female	4004 (80.807%)	2019 (42.336%)	817 (17.932%)	
Male	951 (19.193%)	2750 (57.664%)	3739 (82.068%)	
Habit of exercise				<0.001
No	4091 (82.563%)	3849 (80.709%)	3864 (84.811%)	
Yes	864 (17.437%)	920 (19.291%)	692 (15.189%)	
Drinking status				<0.001
Non or small	4239 (85.550%)	3493 (73.244%)	3043 (66.791%)	
Light	638 (12.876%)	1040 (21.808%)	1110 (24.363%)	
Moderate	78 (1.574%)	236 (4.949%)	403 (8.845%)	
Smoking status				<0.001
Non	3974 (80.202%)	2839 (59.530%)	1938 (42.537%)	
Former	451 (9.102%)	908 (19.040%)	1213 (26.624%)	
Current	530 (10.696%)	1022 (21.430%)	1405 (30.838%)	
NAFLD				<0.001
No	4924 (99.374%)	4329 (90.774%)	2512 (55.136%)	
Yes	31 (0.626%)	440 (9.226%)	2044 (44.864%)	

Values are expressed as n (%) or mean ± SD or median (quartile1-3).

BMI, body mass index; WC, waist circumference; DBP, diastolic blood pressure; SBP, systolic blood pressure; TC, total cholesterol; TG, triglyceride; HDL, high-density lipoprotein cholesterol; GGT gamma-glutamyl transferase; ALT, alanine aminotransferase; AST, aspartate aminotransferase; DBP, diastolic blood pressure; SBP, systolic blood pressure; NAFLD, nonalcoholic fatty liver disease.

### 3.2 Association between TyG-WC and NAFLD

Male, age, BMI, weight, WC, SBP, DBP, ALT, AST, GGT, TC, TG, HbA1C, TyG, were found to be risk factors for NAFLD in the univariate analysis (**Table 2**). A comparison of the effect sizes of TyG-WC on NAFLD in among male, female and all participants is shown in **Table 3**. The Model 1 shows TyG-WC to be positively associated with NAFLD in total participants. According to Model 2, NAFLD risk increased by 1.7% for every unit increase in TyG-WC (OR = 1.017, 95% CI 1.016-1.017, *P*<0.001) in total participants after accounting for age, habit of exercise, smoking status, and SBP. In Model 3, after adjusting for Model 2+ALT, AST, GGT, HDL, TC, TG, FPG, HbA1C, for each unit increase in TyG-WC, the risk of NAFLD increased 1.4% (OR = 1.014, 95% CI 1.013-1.015, *P* < 0.001) in total participants. The fully adjusted OR (95% CI) for NAFLD in total participants was 1.007 (95% CI 1.006–1.009, *P* < 0.001) for every 1-unit increase in TyG-WC.

**Table 2:** Univariate logistics regression model showing variables associated with NAFLD.

	Statistics	OR (95%CI)	*P* value
Gender			
Female	6840 (47.899%)	1.0	
Male	7440 (52.101%)	5.018 (4.513, 5.579)	<0.00001
Age, years	43.533 ± 8.891	1.019 (1.014, 1.024)	<0.00001
BMI, kg/m2	22.068 ± 3.137	1.647 (1.614, 1.681)	<0.00001
Weight, kg	60.283 ± 11.619	1.130 (1.124, 1.136)	<0.00001
WC, cm	76.196 ± 9.100	1.204 (1.195, 1.213)	<0.00001
SBP, mmHg	113.961 ± 14.833	1.053 (1.050, 1.057)	<0.00001
DBP, mmHg	71.141 ± 10.391	1.079 (1.074, 1.084)	<0.00001
ALT, IU/L	19.770 ± 14.459	1.103 (1.098, 1.109)	<0.00001
AST, IU/L	18.227 ± 8.662	1.090 (1.083, 1.097)	<0.00001
GGT, IU/L	19.154 ± 16.165	1.040 (1.037, 1.043)	<0.00001
HDL, mg/dL	56.449 ± 15.472	0.927 (0.923, 0.931)	<0.00001
TC, mg/dL	198.131 ± 33.565	1.013 (1.012, 1.014)	<0.00001
TG, mg/dL	79.030 ± 56.073	1.017 (1.017, 1.018)	<0.00001
HbA1C, %	5.178 ± 0.321	4.417 (3.841, 5.078)	<0.00001
Habit of exercise			
No	11804 (82.661%)	1.0	
Yes	2476 (17.339%)	0.815 (0.724, 0.918)	0.00076
Drinking Status			
Non or small	10775 (75.455%)	1.0	
Light	2788 (19.524%)	0.994 (0.890, 1.109)	0.91106
Moderate	717 (5.021%)	1.152 (0.952, 1.394)	0.14530
Smoking status			
Non	8751 (61.282%)	1.0	
Former	2572 (18.011%)	2.122 (1.904, 2.364)	<0.00001
Current	2957 (20.707%)	1.930 (1.738, 2.144)	<0.00001
TyG-WC	613.431 ± 107.254	1.018 (1.017, 1.018)	<0.00001
TyG	8.012 ± 0.641	7.616 (6.953, 8.344)	<0.00001

**Table 3:** Relationship between TyG-WC and NAFLD risk in different models.

	Model 1 Odds ratios (95%CI)	*P* value	Model 2 Odds ratios (95%CI)	*P* value	Model 3 Odds ratios (95%CI)	*P* value	Model 4 Odds ratios (95%CI)	*P* value
Female								
TyG-WC	1.019 (1.018, 1.021)	<0.001	1.019 (1.017, 1.020)	<0.001	1.016 (1.014, 1.018)	<0.001	1.008 (1.005, 1.011)	<0.001
TyG-WC (trisection)								
T1	1.0		1.0		1.0		1.0	
T2	19.189 (11.753, 31.329)	<0.001	16.388 (9.984, 26.898)	<0.001	10.966 (6.580, 18.274)	<0.001	5.475 (3.243, 9.244)	<0.001
T3	127.822 (78.746, 207.482)	<0.001	94.839 (57.561, 156.260)	<0.001	36.777 (21.339, 63.385)	<0.001	7.162 (3.865, 13.273)	<0.001
*P* for trend	<0.001		<0.001		<0.001			
Male								
TyG-WC	1.016 (1.015, 1.017)	<0.001	1.016 (1.015, 1.017)	<0.001	1.013 (1.012, 1.014)	<0.001	1.007 (1.005, 1.009)	<0.001
TyG-WC (trisection)								
T1	1.0		1.0		1.0		1.0	
T2	8.147 (4.650, 14.274)	<0.001	7.456 (4.250, 13.080)	<0.001	4.467 (2.479, 8.048)	<0.001	2.598 (1.442, 4.679)	0.001
T3	63.144 (36.403, 109.528)	<0.001	52.458 (30.153, 91.262)	<0.001	14.577 (8.070, 26.332)	<0.001	4.292 (2.339, 7.876)	<0.001
*P* for trend	<0.001		<0.001		<0.001		<0.001	
Total								
TyG-WC	1.017 (1.016, 1.018)	<0.001	1.017 (1.016, 1.017)	<0.001	1.014 (1.013, 1.015)	<0.001	1.007 (1.006, 1.009)	<0.001
TyG-WC (trisection)								
T1	1.0		1.0		1.0		1.0	
T2	13.914 (9.614, 20.137)	<0.001	12.147 (8.378, 17.611)	<0.001	7.842 (5.335, 11.527)	<0.001	4.202 (2.845, 6.204)	<0.001
T3	102.810 (71.388, 148.060)	<0.001	80.084 (55.378, 115.812)	<0.001	25.576 (17.236, 37.953)	<0.001	6.534 (4.303, 9.921)	<0.001
*P* for trend	<0.001		<0.001		<0.001			

Model 1, without covariate adjustment; Model 2, adjusted for age, smoking status, habit of exercise, SBP; Model 3, adjusted for Model 2+HbA1C, FPG, TG, TC, HDL, GGT, ALT, AST, and Model 4, adjusted for Model 3+BMI.

Female and male had fully adjusted ORs (95% CI) of 1.008 (1.005, 1.011) and 1.007 (1.005, 1.009), respectively. We also conducted the sensitivity analysis using TyG-WC as a categorical variable (triquantile), and the same trend was observed (*P* for trend was *P* < 0.001).

### 3.3 Results of two-piecewise linear regression model

**Figure 2:**
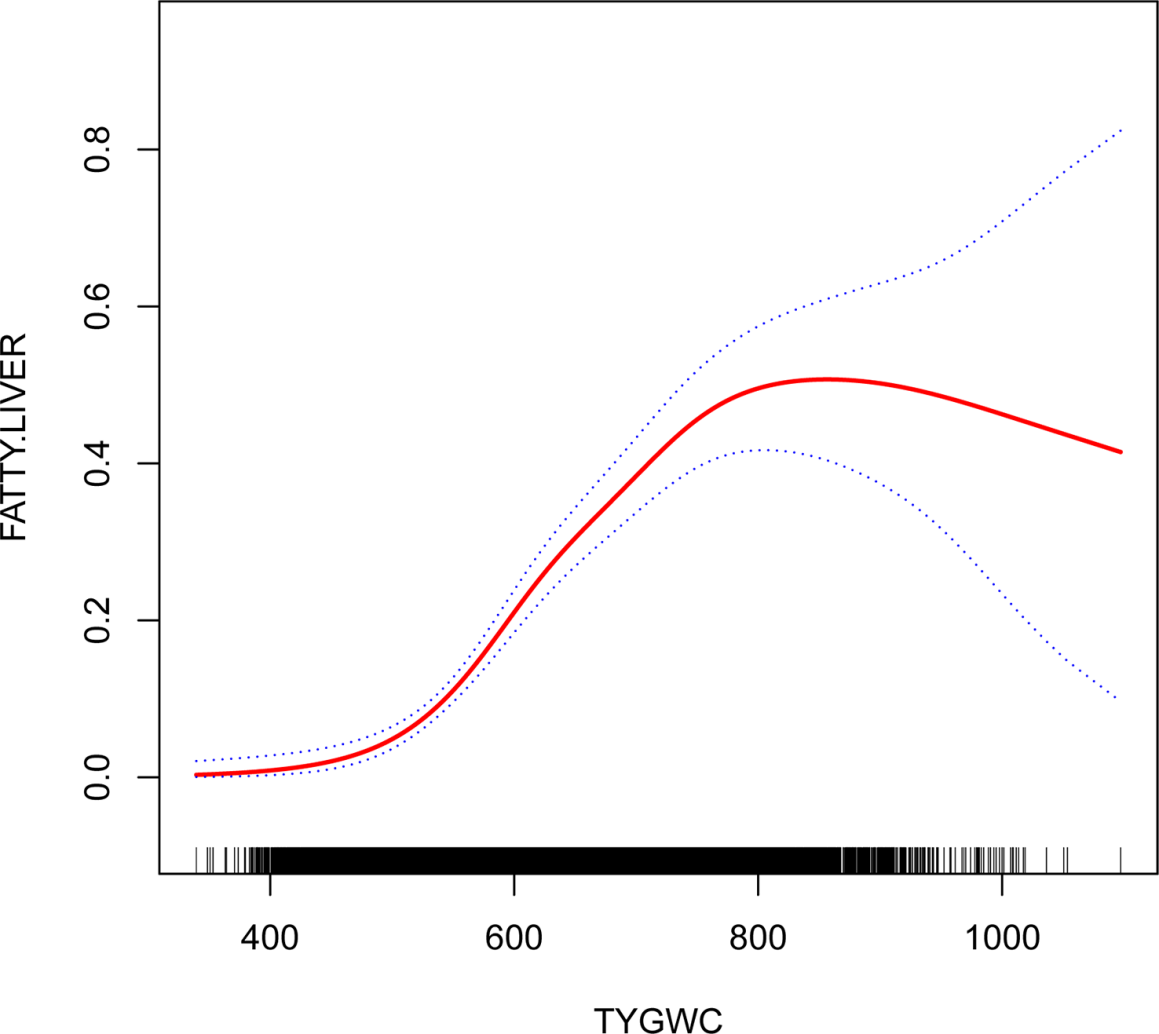
The relationship between triglyceride glucose waist circumference and nonalcoholic fatty liver disease. The graph shows the relationship between TyG-WC and NAFLD. The 95%CI is represented by the area between two dotted lines. When the TyG-WC value is ≤480 or ≥800, there is no correlation with NAFLD. However, when the TyG-WC value is between 480 and 800, there is a significant correlation with NAFLD. As the TyG-WC value increases, so does the risk of NAFLD.

TyG-WC ranging between 480 and 800 showed a significant association between TyG-WC and NAFLD (OR1.009, 95% CI:1.007, 1.010, *P*< 0.001). The risk of NAFLD increased 9% for each additional unit of TyG-WC (**Table 4**; **Figure 2**).

**Table 4:** The results of the two-piecewise linear regression model.

Inflection point of TyG-WC	Odds ratio (OR)	95%CI	*P* value
<480	1.022	0.984-1.062	0.2539
480-800	1.009	1.007, 1.010	<0.0001
>800	1.001	0.996, 1.006	0.7112

Adjusted for age, smoking status, habit of exercise, SBP, HbA1C, FPG, TG, TC, HDL, GGT, ALT, AST, BMI.

### 3.4 Subgroup analysis

In subgroup analyses, there was a significant interaction between BMI and TyG-WC associated NAFLD risk (**Table 5**) (*P* for interaction< 0.001), while the interaction between age, gender, smoking status, drinking status, habit of exercise and TyG-WC was not significant. The results of BMI stratification showed that non-obese people had a higher risk of NAFLD than overweight and obese people.

**Table 5:** Subgroup analysis of the association between TyG-WC on NAFLD risk.

Subgroup	No. of participants	adjusted OR (95% CI)	*P* for interaction
Gender			0.3561
Female	6840	1.008 (1.005, 1.011)	
Male	7440	1.007 (1.005, 1.009)	
Age			0.5639
<45	8319	1.008 (1.006, 1.010)	
>=45, <60	5325	1.008 (1.005, 1.010)	
>=60	636	1.007 (1.000, 1.013)	
BMI			<0.0001
<24	10894	1.010 (1.008, 1.013)	
>=24, <28	2742	1.005 (1.003, 1.007)	
>=28	644	1.004 (0.999, 1.009)	
Drinking status			0.0990
Non or small	10775	1.010 (1.008, 1.012)	
Light	2788	1.004 (1.001, 1.007)	
Moderate	717	1.009 (1.003, 1.015)	
Smoking Status			0.1861
Non	8751	1.009 (1.007, 1.011)	
Former	2572	1.006 (1.003, 1.009)	
Current	2957	1.007 (1.004, 1.010)	
Habit of exercise			0.9967
No	11804	1.007 (1.006, 1.009)	
Yes	2476	1.011 (1.007, 1.015)	

## 4 Discussion

The present study assessed the relationship between TyG-WC and NAFLD among Japanese subjects with normal plasma glucose level. Despite adjusting for other covariates, TyG-WC remained associated with NAFLD in the Japanese population (OR = 1.007, 95% CI 1.006, 1.009). Furthermore, we revealed a threshold effect of TyG-WC and NAFLD, that both low and high levels of TyG-WC had no significant association with NAFLD, but in the range of 480 to 800, TyG-WC was strongly associated with NAFLD. In addition, TyG-WC and BMI interacted to affect NAFLD in subgroup analysis (*P* value for interaction <0.001).

In spite of the complexity of NAFLD’s mechanism, IR plays a crucial role in its progression. The identification of IR, however, is not straightforward. In the current state of IR detection, the gold standard is still the HEC test (27). Due to its complexity and time-consuming nature, HEC is limited in clinical applications. In consequence, the homeostasis model assessment of IR (HOMA-IR) has become a globally recognized alternative indicator of IR. Studies have found an independent relationship between HOMA-IR and NAFLD (28). It is, however, difficult in many laboratories to detect insulin concentrations, which is needed for HOMA-IR to be calculated. As a result, new indicators are needed to identify IR and NAFLD.

There is a growing body of research confirming that TyG can be used for IR assessment, and it has the advantage that it requires just two simple haematological indices (FPG and TG) to calculate. TyG can be used to identify IR through its association with HOMA-IR and HEC (29). It was concluded by Lim et al. that TyG had superior prediction ability over insulin resistance when it came to NAFLD (30). More and more evidence show that obesity is closely related to insulin resistance, and because TyG is universally accepted as a promising surrogate marker of IR, the combination of obesity and TyG may be more powerful than other surrogate markers in identifying IR (14). It has been claimed by Cho et al. that the TyG-WC is an indicator of coronary artery disease that can be used to predict the progression of coronary atherosclerosis better than other indices (31). Khamseh et al. performed a cross sectional study to analyse the association between TyG-WC and NAFLD in individuals with overweight/obesity (20). They concluded that TyG-WC was significant associated with NAFLD in individuals with overweight/obesity. The current study results are similar to theirs. Nevertheless, their study limited its participants to obese and overweight people. Furthermore, the non-linear relationship between TyG-WC and NAFLD was not considered. It was clearly observed in the current study that TyG-WC and NAFLD are not linearly related. NAFLD and TyG-WC ranging between 480 and 800 showed a statistically significant association. The magnitude of the TyG-WC was not associated with NAFLD when it was ≤ 480 or ≥ 800.

Interestingly, our current study found that people with lower BMI had a higher OR value.(BMI <24kg/m^2,OR:1.010, 95%CI:1.008, 1.013)than those with higher BMI(24kg/m^2≤BMI<28kg/m^2,OR:1.005,95%CI:1.003,1.007;BMI≥28,OR:1.004,95%CI:0.999, 1.009). Some studies suggest that the relationship between TyG index and NAFLD risk is significantly stronger in non-obese subjects than in obese subjects (13, 32). It is unclear how BMI influences TyG-WC and NAFLD, but it may be related to relatively lean people’s significantly reduced skeletal muscle mass. Relevant studies have shown that when body mass index decreased, skeletal muscle weight, skeletal muscle index and limb fat decreased significantly, and low muscle mass was positively correlated with NAFLD (33, 34). Further research is needed on the specific mechanism.

### 4.1 Strengths and limitation

The following are some of the strengths of this study (1): A strict adjustment was made for confounding factors in this study (2). Sensitivity analysis was conducted, the continuous independent variables were converted to categorical variables for analysis to improve the reliability of the results (3). It was investigated whether TyG-WC and NAFLD have a nonlinear relationship (4). Different populations were considered when calculating effect sizes.

In spite of this, there are a few limitations to consider (1): The diagnosis of NAFLD in this study was made by ultrasonography rather than liver biopsy. A further limitation of ultrasonography is its inability to distinguish between steatohepatitis and steatosis. Nevertheless, ultrasound examinations have been widely applied in epidemiological studies to diagnose NAFLD (35) (2). Raw data did not include HOMA-IR and waist-to-hip ratio that were associated with NAFLD and IR (3). In this study, nutritional habits and energy intake were not recorded, but covariates associated with dietary habits, such as TG and HDL-C were adjusted (4). The conclusion cannot be generalized to other races since only Japanese subjects were included in the study.

In short, TyG-WC value between 480 and 800 was positively correlated with NAFLD. The magnitude of the TyG-WC was not associated with NAFLD when it was ≤ 480 or ≥ 800. In addition, the effect size was higher in people with lower BMI(BMI <24 kg/m^2) than those with higher BMI (BMI≥24 kg/m^2). Therefore, when TyG-WC≥480 is worthy of attention, especially when TyG-WC is in the range of 480-800, TyG-WC has a strong positive association with NAFLD.

## 5 Conclusions

The relationship between TyG-WC and NAFLD in Japanese subjects with normal plasma glucose level is nonlinear. TyG-WC is positively associated with NAFLD when TyG-WC is ranged between 480 and 800. The magnitude of the TyG-WC is not associated with NAFLD when it is ≤ 480 or ≥ 800. There is potential clinical significance for the TyG-WC in identifying groups at high risk for NAFLD. It is a low-cost and simple and biochemical measurement that can be used to screen and assess NAFLD risk in large populations. Furthermore, these findings could be useful for establishing diagnostic or predictive models of incident NAFLD in the future.

## Data availability statement

The original contributions presented in the study are included in the article. Further inquiries can be directed to the corresponding author.

## Ethics statement

The studies involving human participants were reviewed and approved by Murakami Memorial Hospital’s ethics committee. The patients/participants provided their written informed consent to participate in this study.

## Author contributions

Conceptualization: XJH and XYH. Methodology: XJH. Software: XYH. Validation: YQ. Formal analysis: XYH. Writing—original draft preparation: XYH. Writing—review and editing: YQ and TS. All authors have read and agreed to the published version of the manuscript. All authors contributed to the article.
